# Selected Aspects of Optical Coherence Tomography and Adaptive Optics in Patients with Increased Body Mass Index

**DOI:** 10.3390/biomedicines14020271

**Published:** 2026-01-26

**Authors:** Paulina Szabelska, Dominika Białas, Radosław Różycki, Joanna Gołębiewska

**Affiliations:** Department of Ophthalmology, Military Institute of Aviation Medicine, 01-755 Warsaw, Poland

**Keywords:** body mass index, BMI, optical coherence tomography, OCT, adaptive optics, AO, multimodal imaging

## Abstract

**Background:** The aim of this retrospective study was to evaluate correlations between Optical Coherence Tomography (OCT) and Adaptive Optics (AO) of selected retinal parameters in individuals with increased BMI (≥25.0), including a subgroup analysis for hypertension (HTN). **Methods:** Sixty-three patients (120 eyes) were assessed using AngioVue OCT and rtx1TM AO devices. Retinal thickness (RT), optic nerve head (ONH), ganglion cell complex (GCC), retinal nerve fiber layer (RNFL), and photoreceptor (cone) parameters—density, spacing, regularity, dispersion—were analyzed. **Results:** A negative correlation between BMI and RT in the parafoveal superior and inferior quadrants was observed. Higher BMI was associated with thinner GCC in the superior and nasal parafoveal regions. Additionally, age negatively correlated with cone density and regularity, and positively with cone spacing and dispersion. Numerous correlations were noted between GCC values in OCT and cone parameters in AO, consistent across both HTN and non-HTN subgroups. **Conclusions:** The findings suggested that AO may detect retinal changes earlier than OCT. Multimodal imaging provides valuable insights into early structural changes associated with elevated BMI. Long-term monitoring is recommended to evaluate the progression and clinical impact of these findings.

## 1. Introduction

Body mass index (BMI) is a measure of body fat based on a person’s height and weight. It is widely used to evaluate whether a person is underweight, normal weight, overweight, or obese [1]. Obesity is a complex, multifactorial disease that has a negative impact on health. This chronic condition results from the accumulation of excess body fat within organs, primarily arising due to a prolonged imbalance between the calories consumed and those expended [2]. Obesity is identified when an individual’s BMI is equal or more than 30.0, while overweight, a precursor to obesity, is characterized by a BMI falling between 25.0 and 29.9. Both are linked to a higher mortality rate compared to being normal or underweight, and their prevalence is more widespread than that of underweight individuals [1].

Over the past 50 years, there has been a significant increase in the rate of obesity in the world. This trend is not only continuing but also intensifying, giving rise to an unprecedented epidemic that exhibits no indications of abating in the foreseeable future [3]. It elevates the prevalence of diseases and conditions, i.e., type 2 diabetes mellitus (DMT2), cardiovascular disease (CVD), metabolic syndrome (MetS), hypertension (HTN), non-alcoholic fatty liver disease (NAFLD), cancers, obstructive sleep apnea (OSA), and depression [2].

Despite the well-documented side effects of obesity on the cardiovascular system, there is a lack of comprehensive data regarding its impact on ocular health. Nevertheless, it is acknowledged that elevated BMI can influence various structural and functional aspects of the visual system. Previous studies highlighted its significant consequences on visual functions, including visual acuity (VA) and visual field. Studies also indicated a higher intraocular pressure (IOP), as well as structural changes such as an increased optic disk cavity and thinner layers of retinal nerve fibers (RNFL) in obese individuals [4].

Increased BMI affects the vascular system both morphologically and functionally, potentially inducing endothelial dysfunction and causing damage to the vasculature of the eye. This can result in compromised blood flow and alterations in its structure [5,6]. Due to that fact, obesity has been linked to various eye conditions, including diabetic retinopathy (DR), glaucoma, and age-related macular degeneration (AMD) [7].

Finding screening methods that are economical, non-invasive, and technologically uncomplicated is crucial for the identification and prediction of complications associated with increased BMI. Nowadays, due to expanded availability of multimodal imaging methods in ophthalmology, monitoring the ocular conditions has become easier than before. Optical Coherence Tomography (OCT) is a valuable tool for investigating alterations in the retina, enabling the detection of variations in both thickness and structure. This capability aids clinicians in tracking the disease’s advancement. Spectral Domain OCT (SD-OCT) stands out as a non-invasive imaging technique capable of identifying even the most subtle alterations in layers of the eye structures [8].

Adaptive optics (AO) represents a non-invasive approach to visualize photoreceptors and microcirculation. The rtx1TM, a microscope utilizing AO technology, is equipped with a software specifically designed for image analysis, focused on cones and vessels. AO represents a non-invasive approach to visualize microcirculation and retinal structures, offering a resolution comparable to histological examination and allowing early detection of retinal abnormalities [9,10,11].

Combining imaging methods (OCT and AO) is becoming a useful tool among multimodal imaging in the ophthalmological practice, enabling quick diagnosis of ocular diseases such as glaucoma, DR, or AMD [7,8,9,10]. Due to inconsistent data about ocular abnormalities in patients with increased BMI, we designed this study to assess correlations of OCT and AO findings in this cohort. The aim of the study was to assess the effect of increasing BMI and HTN presence on the retina, including ganglion cell complex (GCC), RNFL, and optic nerve parameters measured by OCT, as well as cone parameters measured by AO, and to identify their correlations.

## 2. Materials and Methods

This retrospective study was conducted between January 2023 and August 2023 at the Department of Ophthalmology of the Military Institute of Aviation Medicine in Warsaw, Poland. The study protocol was approved by the Bioethics Committee at the Military Medical Chamber (approval no. KB 77/2024). Medical records from routine ophthalmological exams in the Outpatient Department of the Military Institute of Aviation Medicine were analyzed. The following data were verified: age, gender, ophthalmological diseases, general health, previous treatment and surgeries, (including bariatric), and previous ophthalmological treatment and surgeries.

The following inclusion and exclusion criteria were applied to the group [Table 1].

Clinical and demographic data were obtained from medical records. To minimize selection bias, all consecutive patients meeting the inclusion criteria during the study period were included. All participants were examined by performing a complete ophthalmological examination with dilated fundus assessment and color fundus photography, OCT and AO.

Best-corrected visual acuity (BVCA) was measured monocularly using LogMAR charts (Lighthouse International, New York, NY, USA) at 5 m. Patients with BVCA = 0.9 and 1.0 were included. Ophthalmological examination was performed using a Topcon SLD701 slit lamp and Volk Superfield lens. Axial length (AL) was measured using the IOL Master 500 (Carl Zeiss Meditec AG, Jena, Germany).

**Figure 1:**
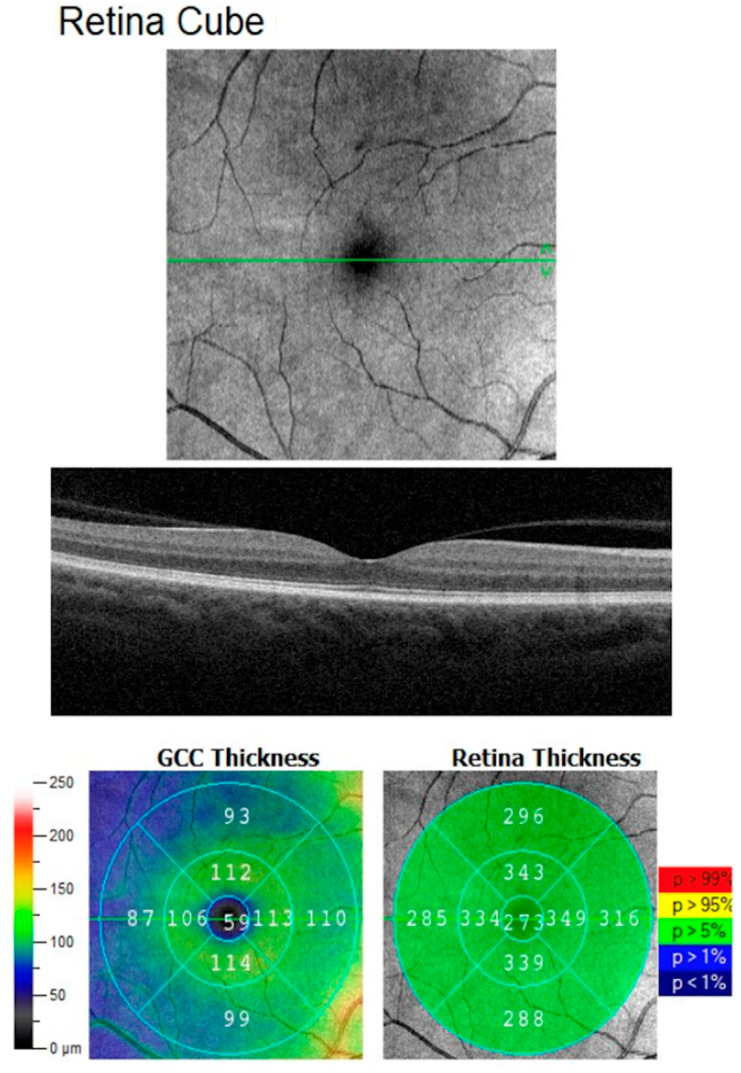
**Representative OCT GCC and RT report of patient’s right eye.** The figure panel shows the following: En Face OCT of macular area, OCT B-Scan, and GCC and RT separately calculated (fovea, temporal, superior, nasal, and inferior parafoveal outcomes were measured), based on the ETDRS contour, and outcomes of quantitative analysis by the software.

OCT examination was performed using the AngioVue Imaging System (Optovue, Inc., Freemont, CA, USA). Macular outcomes were measured in the fovea (0–1 mm) and parafoveally. The parafoveal area, as defined by the 3 mm partial ETDRS grid from the AngioVue software, is the area comprised between the 1–3 mm concentric ring centered of the fovea. The parafoveal area was then further divided into four sectors for quadrant analysis (temporal (T), superior (S), nasal (N), and inferior (I)) (Figure 1).

OCT scans in the HD Retina Cube protocol that covered an area of 6 × 6 mm were performed. Retinal thickness (RT) in the fovea (FT) and parafoveal retinal thickness (PFT) data was obtained from retinal maps. Three scans for each eye were captured, then the best one in quality (with a signal strength index ≥6) was considered for analysis. The quality of the scan (signal strength index) is measured automatically by the device and good quality of the performed scan is a result of 6 or more, on a scale from 0 to 10.

**Figure 2:**
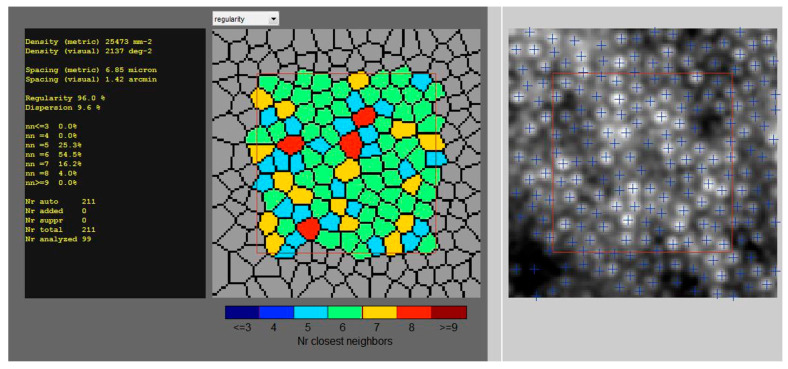
**Representative AO cone report with regularity color map of patient with increased BMI.** Figure panel shows analysis of the cones by the software, including density, spacing, regularity and dispersion, color map of the cone regularity, in vivo picture of the visible cones during examination, and cones marked by blue sign “+”.

AO examination was performed using rtx1TM (Imagine Eyes, Orsay, France). All scans were captured for the four perifoveal areas of the retina, 4° off the center of the fovea (temporally superiorly (TS), temporally inferiorly (TI), nasally superiorly (NS), nasally inferiorly (NI)), with a standardized 80 × 80 μm sampling window size. After finding the foveal reference point of the patient, eccentricities of 4° along the meridians were measured and used for further images analyses (Figure 2). All the scans were repeated three times, and the average results were presented. All the parameters were analyzed using the AOdetect software included with the device.

The data collected from both eyes of the patients studied were taken into analysis.

Additional analysis of patients with co-occurring HTN was performed. All the participants who suffered from HTN had no clinical indications of its ocular complications. Patients were classified as first grade of HTN based on American Heart Association recommendations [12].

### Statistical Analysis

Numerical traits were depicted by their mean, standard deviation, median, and lower-to-upper quartile values. Appropriate corrections for laterality and mixed-effects models were applied. Discrete variables were described by integer numbers and percentages. As stated, when dealing with non-normally distributed or heteroskedastic variables, generalized linear models were fitted. The normality of distribution was assessed using the Shapiro–Wilk W test. Multivariate regressions models were fitted in order to assess the relationships between the investigated traits. The homoskedasticity and normality of data were tested before starting the procedure. Generalized linear models were applied when encountering non-normally distributed variables. The Breusch–Pagan test was performed in order to appraise the homoscedasticity of residuals in the linear regression model. Q-Q plots were auxiliary analyzed in order to assess the normality of the residuals. Due to the fact that the multifactor analyses encompassed the measurements from two eyes per patient, standard error correction consisting in intra-subject correlations was applied. A level of *p* < 0.05 was considered statistically significant. The Pearson correlation coefficient (*r*) was calculated to examine the linear relationship between two continuous variables. There were applied corrections to the Pearson product–moment correlation coefficients. Taking into account the accessibility of specific statistical software, the research team applied the increased precision modality, which is equivalent to the correction for measurement error. Any negative correlation coefficient indicates an inversely proportional relationship (when the independent variable increases, the dependent variable decreases). Any positive correlation coefficient indicates a directly proportional relationship (when the independent variable rises, the dependent variable also does). NS stands for ‘not significant’. According to relevant data, Authors decided not to apply multiple testing. All the statistical procedures were performed by using Statistica 13.3 (TIBCO Software Inc., Palo Alto, CA, USA).

## 3. Results

A total of 63 adults (120 eyes) with BMI ≥ 25.0 was included in the study. A total of 34.92% (*n* = 22) of participants was female and 65.08% (*n* = 41) of participants were male. In terms of age, participants were all equal or more than 18 years old. A total of 18 of the individuals was diagnosed with HTN (well-treated, no more than first grade). In some cases, only one eye of the patient met the requirements of the study, based on the eligibility criteria for participation (Table 1).

Table 2 and Table 3 show the in-depth descriptive characteristics of the study cohort.

We have used multivariate regression models to analyze the relationships of the retinal parameters with age, BMI, and prevalence of HTN.

FT and PFT are presented in Table 4. Among the parafoveal quadrants, the lowest thickness of retina was presented in temporal quadrant. We have found a negative correlation between BMI and RT in parafoveal superior and inferior quadrants (the higher BMI value, the thinner retina), which were statistically significant (respectively: *p* = 0.0252 and *p* = 0.0359). In nasal and temporal quadrants, differences were not statistically significant. There were no correlations between BMI patients’ age and prevalence of HTN and RT in the study groups.

In Table 5, we presented results of the optic nerve head parameters. We found no statistically significant correlations between the age, BMI and HTN presence, and optic nerve parameters (including disk area and cup-to-disk ratio).

GCC results are shown in Table 6. The GCC in parafoveal superior and nasal quadrants was thinner while the BMI value increased (*p* value was, respectively, 0.0148 and 0.0318). In the remaining GCC quadrants, there was no correlation with BMI. We found no correlations between GCC and age and HTN presence.

RNFL results are presented in Table 7. RNFL was thinner in temporal and nasal quadrants than in superior and inferior. RNFL in the superior quadrant was positively correlated with higher BMI values (*p* = 0.0018). We found no statistically important correlations with BMI and RNFL in other quadrants. There were no statistically significant correlations between RNFL and RT, and GCC and HTN presence.

We measured choroidal thickness (CT) in the group and the results were as follows: Mean = 301.00 ± 55.36 μm and Median = 298.00 μm (267.00–343.00). We found negative correlation with CT and age, r = −0.40 (*p* < 0.0001). We also found no correlation with BMI and prevalence of HTN.

The results of AO obtained for cone parameters including density, spacing, regularity and dispersion were comparable between quadrants and are presented in Table 8.

We have noted statistically significant negative correlation between cone density and age (Pearson correlation). The results are as follows:

- In TS quadrant r = −0.54 (*p* < 0.0001);
- In TI quadrant r = −0.44 (*p* < 0.0001);
- In NS quadrant r = −0.49 (*p* < 0.0001);
- In NI quadrant r = −0.52 (*p* < 0.0001).

We found positive correlation (Pearson correlation) between cone spacing and dispersion and patients’ age in all the quadrants; all of them were statistically significant. The results are as follows:

- In TS quadrant: spacing r = 0.50 (*p* < 0.0001), dispersion r = 0.39 (*p* = 0.0002);
- In TI quadrant: spacing r = 0.41 (*p* < 0.0001), dispersion r = 0.42 (*p* = 0.0002);
- In NS quadrant: spacing r = 0.52 (*p* < 0.0001), dispersion r = 0.37 (*p* = 0.0017);
- In NI quadrant spacing r = 0.49 (*p* < 0.0001), dispersion r = 0.36 (*p* = 0.0048).

Negative correlation (Pearson correlation) between age and cone regularity were statistically significant in almost all quadrants, despite NI. The results are as follows:

- In TS quadrant r = −0.29 (*p* = 0.0053);
- In TI quadrant r = −0.37 (*p* = 0.0017);
- In NS quadrant r = −0.30 (*p* = 0.0057);
- In NI quadrant was not statistically significant.

BMI and cone density were positively correlated in TS and NS quadrants (r = 0.19 (*p* = 0.0031) and r = 0.16 (*p* = 0.0131), respectively).

**Figure 3:**
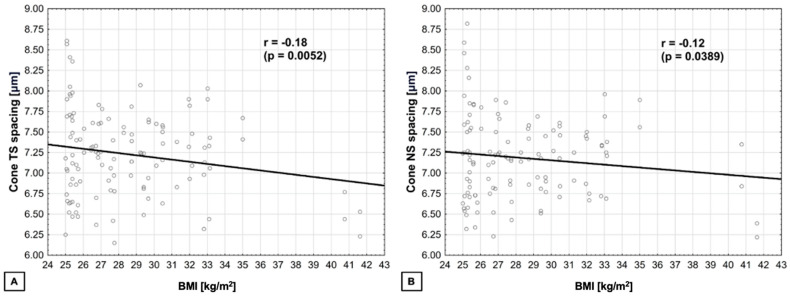
**BMI and cone spacing correlations.** (**A**) BMI and cone spacing in TS quadrant—negative; (**B**) BMI and cone spacing in NS quadrant—negative.

BMI and cone spacing were negatively correlated in TS and NS quadrants (r = −0.18 (*p* = 0.0052) and r = −0.12 (*p* = 0.0389), respectively) (Figure 3).

**Figure 4:**
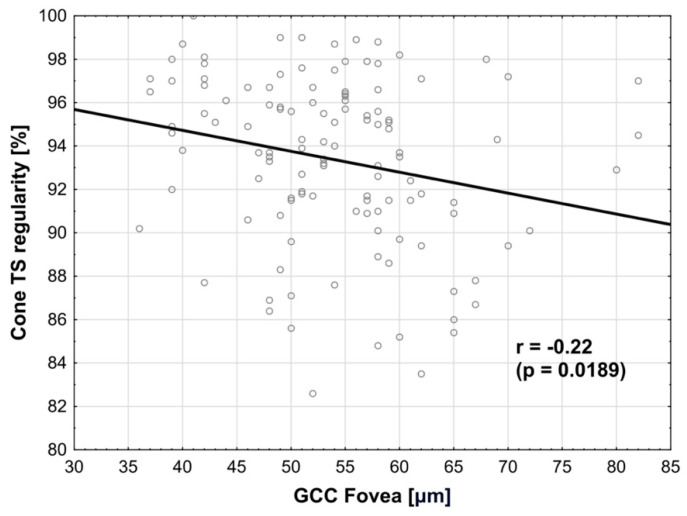
**Correlation between cone regularity in TS quadrant and GCC in the fovea.** Negative correlation is observed.

**Figure 5:**
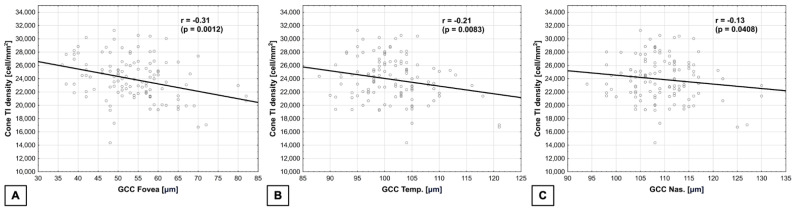
**Correlations between (multivariate regression) cone density in TI quadrant and GCC** (**A**) In the fovea—negative. (**B**) In the temporal quadrant—negative. (**C**) In the nasal quadrant—negative.

**Figure 6:**
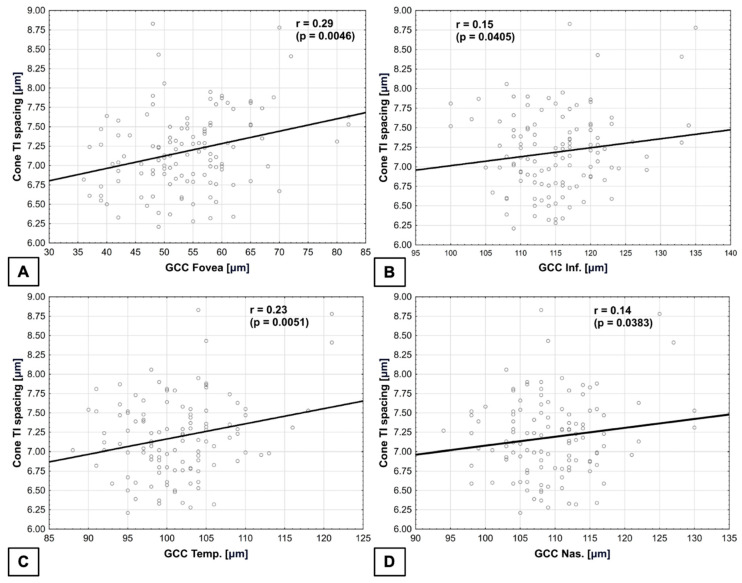
**Correlations between cone spacing in TI quadrant and GCC.** (**A**) In the fovea—positive. (**B**) In the inferior quadrant—positive. (**C**) In the temporal quadrant—positive. (**D**) In the nasal quadrant—positive.

**Figure 7:**
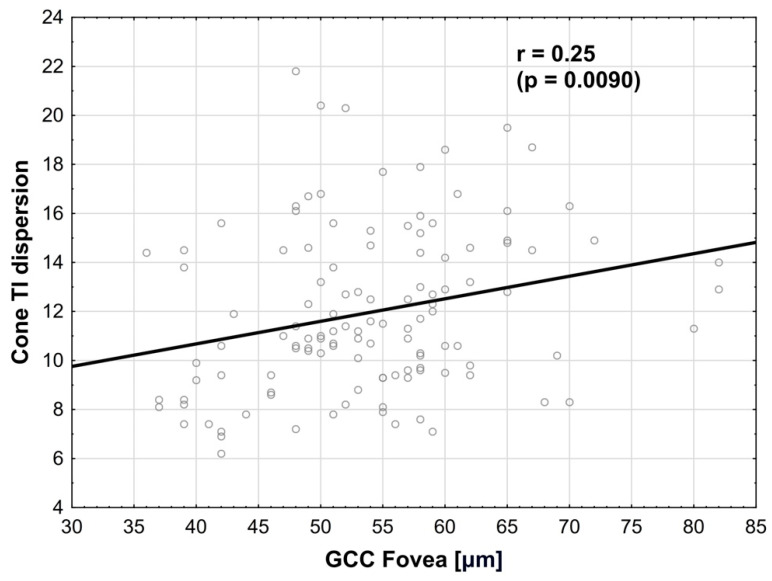
**Correlation between cone regularity in TI quadrant and GCC in the fovea.** Positive correlation is observed.

**Figure 8:**
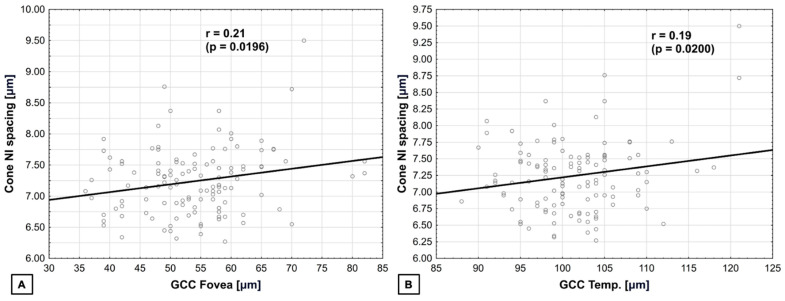
**Correlation between cone spacing in NI quadrant and GCC.** (**A**) In the fovea—positive. (**B**) In the temporal quadrant—positive.

**Figure 9:**
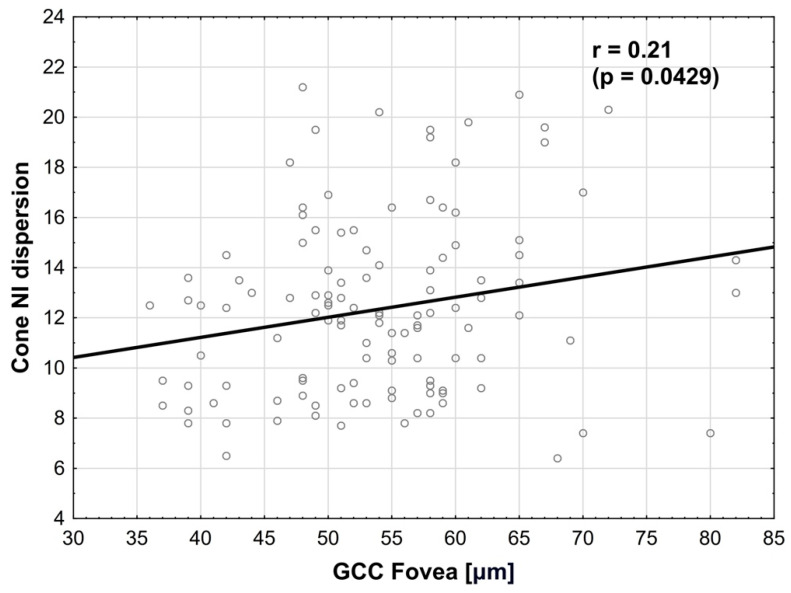
**Correlation between cone dispersion in NI quadrant and GCC in the fovea**. Positive correlation is observed.

We performed multivariate regression models between GCC (OCT) and cone characteristics (AO) and found following correlations:

- Cone regularity in TS quadrant was negatively correlated with GCC in the fovea, where r = −0.22 (*p* = 0.0189) (Figure 4). The normality test of residuals (i.e., the Q-Q plot) did not reveal any significant deviations from normality in the model used.

- Cone density in TI quadrant was negatively correlated (multivariate regression) with GCC in the fovea, r = −0.31 (*p* = 0.0012), as well as in the temporal quadrant, where r = −0.21 (*p* = 0.0083), and in the nasal quadrant, where r = −0.13 (*p* = 0.0408) (Figure 5). The normality test of residuals did not reveal any significant deviations from normality in the model used.

- Cone spacing in TI quadrant was positively correlated with GCC in the fovea, where r = 0.29 (*p* = 0.0046), as well as with GCC in the inferior quadrant, where r = 0.15 (*p* = 0.0405). It is also positively correlated with GCC in the temporal quadrant, where r = 0.23 (*p* = 0.0051), and GCC in the nasal quadrant, where r = 0.14 (*p* = 0.0383) (Figure 6). The normality test of residuals did not reveal any significant deviations from normality in the model used.

- Cone dispersion in TI quadrant was positively correlated (multivariate regression) with GCC in the fovea, where r = 0.25 (*p* = 0.0090) (Figure 7). The normality test of residuals did not reveal any significant deviations from normality in the model used.

- Cone spacing in NI quadrant was positively correlated (multivariate regression) with GCC in the fovea, where r = 0.21 (*p* = 0.0196), as well as with GCC in the temporal quadrant, where r = 0.19 (*p* = 0.0200) (Figure 8). The normality test of residuals did not reveal any significant deviations from normality in the model used.

- Cone dispersion in NI quadrant was positively correlated (multivariate regression) with GCC in the fovea, where r = 0.21 (*p* = 0.0429) (Figure 9). The normality test of residuals did not reveal any significant deviations from normality in the model used.

## 4. Discussion

Retinal morphology can be assessed in detail by multimodal imaging including techniques such as OCT and AO, and represent potent and synergistic methodologies. Each has profoundly transformed the field of retinal and optic nerve head (ONH) imaging, offering researchers the capability to routinely observe cellular-level details in either cross-sectional or En Face perspectives. Moreover, integrating conventional clinical imaging with findings from these complementary techniques enhances our comprehensive comprehension of the anatomy being studied [13]. OCT provides measurements not only of the retina but also of the choroid, which may be the predictors of an increased risk of concomitant or future vascular pathologies in obese patients [14,15,16]. The advancement in AO technology has enhanced the optical systems’ resolution to 2 µm by rectifying optical wave-front aberrations. This innovation has significantly transformed the approaches for studying eye structures in vivo [17]. AO-based retinal imaging has been proven to be valuable in monitoring conditions such as glaucoma, AMD, DR, or central serous chorioretinopathy (CSCR).

In this research, we conducted non-invasive assessments of RT, GCC, and RNFL using OCT, and examined cone morphology using rtx1 AO within a cohort of individuals with elevated BMI.

In our study, FT and PFT were measured. We noted a negative correlation between BMI and RT in parafoveal superior and inferior quadrants, which can be explained by “horizontal–vertical anisotropy” [18]. This term refers to differences in visual perception across horizontal and vertical axes. It has been observed that our visual system exhibits a higher concentration of cone photoreceptors along the horizontal meridian, likely due to the predominant use of our horizontal retina when engaged in tasks such as reading. This theory has been explored in psychophysical studies, which demonstrated that, at a given distance from the center of vision, our ability to perceive, contrast, and resolve spatial details is superior along the horizontal axis compared to the vertical one. These findings have been further corroborated in studies involving AO among a control group comprising healthy individuals [19]. This phenomenon may explain why RT in the superior and quadrants decreases earlier than nasal and temporal due to pathological changes in the retina. In contrast to our results, Salehi MA et al. found five studies in which RT has been examined in patients with increased BMI (in total: 272 cases and 197 controls). In their meta-analysis, no significant differences between BMI and RT were observed compared to healthy individuals [8].

Quantitative characteristics at the cellular level pertaining to retinal ganglion cells (GCs) hold the potential to serve as crucial biomarkers, enhancing the diagnosis and monitoring of neurodegenerative conditions such as glaucoma, Parkinson’s disease, and Alzheimer’s disease [20]. Nevertheless, the axons originating from the temporal retina follow a curving path around the macula, creating the papillomacular beam. Preserving this beam is critical for vision since the axons originate from the fovea and extend towards the temporal edge of the ONH. This ensures the continuity of central vision, as a minimum thickness of nerve fibers in the temporal ON is maintained, making this localization the most well-preserved [21]. Therefore, the assessment of GCC in the temporal quadrant in patients with increased BMI seems to be crucial. In our study, GCC in parafoveal superior and nasal quadrants were thinner while the BMI value increased, which may confirm that patients with a higher BMI may be more susceptible to the previously mentioned neurodegenerative diseases and the temporal part is supposed to be damaged later.

Glaucoma stands as the primary cause of irreversible blindness on a global scale, marked by the gradual impairment and depletion of GCC and RNFL. Numerous investigations have demonstrated the application of OCT and AO in the context of glaucoma [19,22,23,24,25,26,27,28,29,30]. Several research inquiries have delved into the impact of glaucoma on photoreceptors, yet the findings exhibit inconsistencies. Researchers have illustrated alterations in the structural integrity of cones, accompanied by diminished visual sensitivity, through AO images [24]. Other studies presented changes in cone morphology in individuals exhibiting visual field defects resembling those seen in glaucoma [25]. Contrary to that, Hasegawa and colleagues observed no distinctions in either cone density or spatial arrangement in eyes affected by glaucoma [26].

The available literature suggested a clear correlation between aging and progressive thinning of the RNFL, accompanied by a loss of GCC. Age-related RNFL thinning follows a characteristic topographic distribution. Typically, the peripapillary RNFL exhibits its greatest thickness in the inferior rim, followed by the superior, nasal, and finally the temporal rim, which is the thinnest. This characteristic arrangement, referred to as the inferior–superior–nasal–temporal (ISNT) pattern, reflects the anatomical convergence of nerve fibers from the superior and inferior arcuate bundles to the ONH [21].

Our findings are consistent with these age-related changes described in the literature. In the studied group, RNFL thickness was lower in the temporal (T) and nasal (N) quadrants compared to the superior (S) and inferior (I) quadrants, which supports the presence of the ISNT pattern and aligns with previously reported aging-associated RNFL thinning.

Interestingly, RNFL in superior quadrant was positively correlated with higher BMI values and that requires future research. We found no statistically important correlations with BMI and RNFL in other quadrants. Recently, there have been reports indicating a connection between a lower RNFL thickness and higher BMI in a group of adult individuals [31]. Furthermore, Pacheco–Cervera et al.’s research has demonstrated that the reduction in RNFL thickness occurred in the inferior, superior, and nasal quadrants in children with higher BMI. Interestingly, the temporal quadrant remained unaffected in severely obese children [21].

AO imaging primarily focuses on assessing key parameters of photoreceptors, including cone density, spacing, dispersion, and regularity. To estimate density, the number of cones is divided by the corresponding area. Cone spacing involves measuring the distances between adjacent cells. Dispersion was characterized as the spread of cones, quantified by the coefficient of variation in cone spacing. Given that cones typically form a hexagonal lattice pattern, cone regularity is determined by the frequency of cones having precisely six adjacent cells [32,33]. We can also measure total cone spacing and assess all types of cone cell regularity. We found that BMI and total cone spacing were negatively correlated in NS as well as in TS quadrant and that is why we can look for initial pathological changes in this area. We noted that the cone spacing in TI quadrant was positively correlated with GCC in the fovea, inferior, temporal and nasal quadrants. We described a similar correlation in cone spacing in the NI quadrant and GCC in the fovea and temporal quadrant, which suggests that the initial changes can be visible earlier over the larger area (more quadrants) in AO than in OCT. On the other hand, we noted that BMI and cone density was positively correlated in TS and NS quadrants. Cone density was higher when GCC decreases in the fovea, temporal and nasal quadrants, which can be the subject of further research as we did not find confirmation in the available data. In contrast to our results, Zaleska–Żmijewska et al. in their study found no differences in cone density and regularity at different BMI levels and suggested that cone morphology is not affected by weight in healthy eyes [34].

Our study encompassed individuals with previously diagnosed and effectively managed HTN, limited to no more than the first grade. We observed no statistically significant variances in RT, GCC, RNFL, and cone morphology between the subgroups with and without HTN, which suggests that retinal abnormalities due to HTN occur in more advanced stages of HTN.

Consequently, our imaging with OCT was minimally affected and AO examination indicated early or potentially pre-disease states of those changes which occurred with age. In the current study, we confirm the hypothesis that age is associated with abnormalities of the cone photoreceptor layer in higher BMI patients as revealed on AO. We found negative correlation between cone density and age, positive correlation between cone spacing and dispersion, as well as negative correlation between age and cone regularity (despite NI quadrant). On average, the density of cones tends to decrease as age increases. However, there is significant variability, with both younger and older subjects [35,36]. That was confirmed in our study.

It is important that cone changes in AO were noted in other ophthalmic diseases than glaucoma such as DM and CSCR. Lombardo and colleagues observed a reduction in cone number in AO in eyes with DM compared to control groups. They also found loss of cones in individuals with glucose intolerance, elevated glycohemoglobin levels, and indications of DR [37]. Ochinciuci et al. demonstrated that photoreceptor density was notably lower in eyes affected by CSCR when compared to healthy eyes [38]. Photoreceptor density decrease in older patients may confirm that they are more susceptible to ophthalmological complications in the course of ophthalmic diseases such as DR or CSCR.

Although OCT boasts excellent axial resolution, its lateral resolution is restricted to >20 microns owing to the eye’s monochromatic aberrations [39]. Conversely, AO, through the correction of these aberrations, enables the En Face evaluation of individual photoreceptor cells [40,41]. That confirms the fact that visibility of the photoreceptor layer can be better assessed by AO than by OCT.

To the best of our knowledge, this is the first study which assesses correlations between OCT parameters and photoreceptors parameters in AO in patients with higher BMI. This is also the first study which assesses cone parameters in AO, in which male participants were included. The obtained results suggest that morphological changes in the retina visible in adaptive optics among these patients may appear earlier than changes in OCT. Investigation of a larger group of patients is needed to confirm it.

### 4.1. Limitations

The research was carried out on a limited number of participants, and the evaluation of alterations in specific parameters focused exclusively on the influence among individuals with an elevated BMI, without including a group with a BMI below 25.0. In our research comparing subgroups with and without HTN, the subgroup with HTN (well-treated, no more than first grade) comprised only 18 patients.

### 4.2. Strengths

The groups were selected considering parameters that could disrupt the interpretation of results, such as patients’ refractive error, axial length, and visual acuity. We used automated measurements to eliminate any potential errors associated with manual methods. In the assessment of the retina, we utilized multimodal imaging, including OCT and AO, to thoroughly evaluate retinal (especially photoreceptor) changes occurring in patients with higher BMI. As part of the study, an analysis of retinal parameters was conducted not only based on the BMI value, but also on the coexistence of HTN in this group of patients.

### 4.3. Clinical and Research Implications

Our findings suggest that AO imaging can detect early retinal changes in patients with elevated BMI that may not yet be visible on OCT, highlighting its potential as a sensitive tool for early monitoring. For clinicians, this emphasizes careful assessment of paracentral and temporal regions; for researchers, it underscores the value of combining AO and OCT to study retinal structure and its relation to BMI.

## 5. Conclusions

The observation of retinal structure is essential for comprehending the pathophysiology of eye-related conditions and plays a crucial role in monitoring their progression and the impact of treatments.

Increased BMI affects the retina both morphologically and functionally and this can result in compromised alterations in its structure which is visible in multimodal imaging. Those methods containing OCT and AO, used simultaneously during examination, can help in the diagnostic process as simple and non-invasive techniques to identify early signs of increased BMI ocular complications. They may be used to monitor the early retinal abnormalities in patients with increased BMI and to identify potentially pre-disease states.

Our study confirms that the interpretation of OCT may be completed by other multimodal imaging methods, such as AO, and pathological changes can be visible earlier in AO. Future research should try to explain the causes of processes occurring in patients with higher BMI and multi-morbidities.

## Figures and Tables

**Table 1:** Participants—inclusion and exclusion criteria.

Inclusion Criteria:	Exclusion Criteria:
Age 18 or more BMI equal or more than 25.0 No ophthalmological diseases or injuries in medical history No ophthalmological treatment performed in the past No chronic diseases diagnosed, despite well-treated hypertension (no more than I grade)	Poor quality of OCT scans <6 Poor quality of AO scans, inability to assess cones and vessels in AO images Ophthalmological disease diagnosed during examination Best visual corrected acuity (BVCA) (≤0.8 LogMar) Miopia or hyperopia (>−3.0 Dsph or >+3.0 Dsph) Axial length (AL) <22.0 mm or >24.0 mm IOP > 21 mmHg

**Table 2:** Baseline characteristics of the study group (discrete variables) (n = 63 individuals).

Analyzed Trait	N [%]
No. of participants, n [%]	63 (n/a)
No. of eyes, n [%]	120 (n/a)
Gender:	
- Female	22 (34.92)
- Male	41 (65.08)
Diagnosis of arterial hypertension	18 (28.57)
Smoking status:	
- Non-smoker	47 (74.61)
- Current smoker	2 (3.17)
- Ex-smoker (at least 5 years)	14 (22.22)
Body silhouette:	
- Overweight	47 (74.60)
- 1st degree obesity	13 (20.64)
- 2nd degree obesity	3 (4.76)

**Table 3:** Baseline characteristics of the study group (numerical variables) (n = 63 individuals).

Analyzed Trait	Statistical Parameter *			
	M	SD	Me	Q_1_–Q_3_
Age [years]	50.56	15.41	57.00	36.50–64.00
Body Mass Index [kg/m^2^]	28.42	3.60	27.43	25.39–30.30

* Statistical measures used: M—mean; SD—standard deviation; Me—median; Q—quartiles.

**Table 4:** Descriptive statistics for the foveal and parafoveal thicknesses [µm] (n = 120 eyes).

Thickness [µm]	Statistical Parameter *			
	M	SD	Me	Q_1_–Q_3_
Foveal	257.77	20.70	259.50	242.00–270.00
Parafoveal, S	329.88	12.32	328.00	321.50–338.00
Parafoveal, I	327.08	11.93	325.00	319.00–336.00
Parafoveal, T	316.03	12.30	314.00	306.00–327.50
Parafoveal, N	329.42	13.36	327.00	321.00–339.00

* Statistical measures used: M—mean; SD—standard deviation; Me—median; Q—quartiles. S—superior, I—inferior, T—temporal, N—nasal.

**Table 5:** Descriptive statistics for the optic nerve head [μm] (n = 120 eyes).

Analyzed Trait	Statistical Parameter *			
	M	SD	Me	Q_1_–Q_3_
Disk area [mm^2^]	1.97	0.33	1.94	1.77–2.14
Cup-to-disk ratio	0.2958	0.1449	0.3050	0.1900–0.4000

* Statistical measures used: M—mean; SD—standard deviation; Me—median; Q—quartiles.

**Table 6:** Descriptive statistics for the ganglion cell complex [µm] (n = 120 eyes).

Ganglion Cell Complex [µm]	Statistical Parameter *			
	M	SD	Me	Q_1_–Q_3_
Foveal	53.88	9.12	54.00	48.00–59.00
Parafoveal, S	112.96	6.06	113.00	109.00–116.00
Parafoveal, I	114.97	6.56	115.00	110.00–119.00
Parafoveal, T	101.16	6.03	101.00	97.00–104.00
Parafoveal, N	109.38	6.41	108.50	10.500–113.00

* Statistical measures used: M—mean; SD—standard deviation; Me—median; Q—quartiles. S—superior, I—inferior, T—temporal, N—nasal.

**Table 7:** Descriptive statistics for the retinal nerve fiber layer [μm] (n = 120 eyes).

RNFL [μm]	Statistical Parameter *			
	M	SD	Me	Q_1_–Q_3_
Superior	114.32	14.26	113.50	105.50–125.00
Inferior	122.78	18.36	120.50	109.00–136.50
Temporal	65.05	7.89	64.50	60.00–70.00
Nasal	79.26	12.89	79.00	70.00–88.50

* Statistical measures used: M—mean; SD—standard deviation; Me—median; Q—quartiles.

**Table 8:** Descriptive statistics for the cone measurements (n = 120 eyes).

Quadrant	Cone Parameter	Statistical Parameter *			
		M	SD	Me	Q_1_–Q_3_
TS	Density	23,617	3259	23,100	21,419–25,810
	Spacing	7.23	0.51	7.25	6.86–7.59
	Regularity	93.38	3.88	93.95	91.00–96.50
	Dispersion	11.88	2.75	11.25	9.90–13.60
TI	Density	23,907	3235	23,792	21,638–26,105
	Spacing	7.19	0.51	7.15	6.83–7.51
	Regularity	93.83	4.21	94.80	91.50–96.75
	Dispersion	11.96	3.33	11.25	9.40–14.50
NS	Density	23,784	3224	23,629	21,376–26,354
	Spacing	7.18	0.50	7.19	6.79–7.52
	Regularity	93.57	4.22	94.70	90.85–96.70
	Dispersion	11.58	2.96	10.70	9.30–13.70
NI	Density	23,622	3129	23,380	21,563–25,900
	Spacing	7.24	0.54	7.21	6.85–7.53
	Regularity	93.64	4.19	93.90	90.85–97.15
	Dispersion	12.33	3.52	12.10	9.30–14.35

* Statistical measures used: M—mean; SD—standard deviation; Me—median; Q—quartiles. TS—temporal superior, TI—temporal inferior, NS—nasal superior, NI—nasal inferior.

## Data Availability

The original contributions presented in this study are included in the article. Further inquiries can be directed to the corresponding author.
